# Engine-Operating Load Influences Diesel Exhaust Composition and Cardiopulmonary and Immune Responses

**DOI:** 10.1289/ehp.1003101

**Published:** 2011-04-25

**Authors:** Jacob D. McDonald, Matthew J. Campen, Kevin S. Harrod, JeanClare Seagrave, Steven K. Seilkop, Joe L. Mauderly

**Affiliations:** 1 Lovelace Respiratory Research Institute, Albuquerque, New Mexico, USA; 2 SKS Consulting Services, Siler City, North Carolina, USA

**Keywords:** diesel exhaust, modeling, mouse, particulate matter, viral

## Abstract

Background: The composition of diesel engine exhaust (DEE) varies by engine type and condition, fuel, engine operation, and exhaust after treatment such as particle traps. DEE has been shown to increase inflammation, susceptibility to infection, and cardiovascular responses in experimentally exposed rodents and humans. Engines used in these studies have been operated at idle, at different steady-state loads, or on variable-load cycles, but exposures are often reported only as the mass concentration of particulate matter (PM), and the effects of different engine loads and the resulting differences in DEE composition are unknown.

Objectives: We assessed the impacts of load-related differences in DEE composition on models of inflammation, susceptibility to infection, and cardiovascular toxicity.

Methods: We assessed inflammation and susceptibility to viral infection in C57BL/6 mice and cardiovascular toxicity in APOE^–/–^ mice after being exposed to DEE generated from a single-cylinder diesel generator operated at partial or full load.

Results: At the same PM mass concentration, partial load resulted in higher proportions of particle organic carbon content and a smaller particle size than did high load. Vapor-phase hydrocarbon content was greater at partial load. Compared with high-load DEE, partial-load DEE caused greater responses in heart rate and T-wave morphology, in terms of both magnitude and rapidity of onset of effects, consistent with previous findings that systemic effects may be driven largely by the gas phase of the exposure atmospheres. However, high-load DEE caused more lung inflammation and greater susceptibility to viral infection than did partial load.

Conclusions: Differences in engine load, as well as other operating variables, are important determinants of the type and magnitude of responses to inhaled DEE. PM mass concentration alone is not a sufficient basis for comparing or combining results from studies using DEE generated under different conditions.

Advances in engine, fuel, and after-treatment technologies such as particle traps have markedly reduced emissions from recent model diesel engines, but some older engines remain in use, and diesel engine exhaust (DEE) has likely contributed to associations between health and proximity to heavy traffic (reviewed by Boothe and Shendell 2008). Potential cancer risks remain of interest, but studies involving experimental exposures of humans and animals now focus predominantly on a broad range of noncancer effects (Hesterberg et al. 2009; Mauderly and Garshick 2009).

Variations in the composition of DEE can influence biological responses (McDonald et al. 2004c; Stevens et al. 2009). The engines, fuels, and the operating modes of engines used for experimental exposures vary widely among laboratories; in fact, no “standard” exists for experimental DEE. Neither is there a standard for describing the exposure atmospheres; the detail with which exposures are characterized varies considerably, and most comparisons among studies are made on the basis of particulate matter (PM) mass concentrations. Although long recognized among the engineering community, the substantial variation in DEE among operating conditions and its potential impact on comparability among studies has received only modest acknowledgment among the health research community.

Hypotheses concerning the “toxic” components of DEE have historically focused on the PM component and include associations with carbon, particle size, particle number, particle surface area, and mass. DEE also consists of variable amounts of nitrogen oxides, sulfur oxides, and organic gases and vapors. Indeed, PM is typically a minor part of the total mass emissions of even older engines (McDonald et al. 2004b). The composition of DEE varies with differences in engine performance, engine characteristics (e.g., size, fuel injection strategy), fuel and lubricant type, engine load, environmental conditions, and after-treatment devices (Hesterberg et al. 2009; Lloyd and Cackette 2001). Engine load alone can affect the ratio of organic to elemental carbon in diesel PM, with a higher organic:elemental ratio at low loads (Shi et al. 2000).

Although some experimental exposures of humans to DEE have used engines operated on realistic variable-duty cycles (e.g., Barath et al. 2010), most have used engines operated in steady-state modes. For example, Umeå University in Sweden conducted studies with healthy volunteers who inhaled exhaust from an idling engine in a laboratory setting. The results of these investigations showed increased airway inflammation and reactivity (Rudell et al. 1996), reduced forearm blood flow in response to vasodilators and fibrinolytic dysfunction (Mills et al. 2005; Törnqvist et al. 2007), increased *ex vivo* thrombus formation (Lucking et al. 2008), increased arterial stiffness (Lundbäck et al. 2009), and changes in the electrical activity in the brain (Crüts et al. 2008), but no significant change was observed in *ex vivo* fibrin clot formation (Metassan et al. 2010). That laboratory also showed ischemic alterations of the electrocardiogram (ECG) of men with previous myocardial infarctions (Mills et al. 2007). In a study conducted at the University of Washington in a human exposure laboratory, researchers found that exhaust from an engine operated in steady state at 75% load altered gene expression in circulating monocytes (Peretz et al. 2007) and caused peripheral vasoconstriction and increased circulating vasoactive factors (Peretz et al. 2008b) but did not significantly affect blood coagulation factors (Carlsten et al. 2008), prothrombotic markers (Carlsten et al. 2008), or heart rate (HR) abnormalities (Peretz et al. 2008a). Because none of the above publications provided detailed descriptions of the DEE, it is difficult to determine how differences in engine load may have influenced composition and the comparability of results among laboratories or whether the same outcomes might result from exposure to DEE emitted from vehicles during typical on-road use.

We conducted the present study to determine the influence of engine-operating conditions on selected responses of mice exposed to DEE by inhalation. In particular, we investigated the influence of engine work (load) under two different operating conditions. We measured indices of lung oxidative stress and inflammatory potential, susceptibility to lung infection, and alterations in HR and HR abnormalities because these parameters had been found at the Lovelace Respiratory Research Institute to be affected by DEE at the PM concentration used in the present study (Campen et al. 2003; Harrod et al. 2003; Seagrave et al. 2005). As expected, the composition of DEE differed at high and low engine loads. High engine load caused greater lung responses than did low engine load, but varying engine load did not affect HR responses.

## Materials and Methods

We compared responses to DEE under the two engine load conditions at the same PM mass concentrations, which ranged from 250 to approximately 3,500 μg/m^3^. These concentrations span PM concentrations of DEE that may exist in high ambient to occupational exposure in humans (McDonald et al. 2002). We measured biological responses and DEE composition identically under the two conditions.

*Exposure.* The exposure system has been described (McDonald et al. 2004a) and is briefly summarized here. DEE was produced by a 5,500-W single-cylinder diesel generator (model YDG 5500E; Yanmar, Osaka, Japan) that contained a 406-cc–displacement air-cooled engine. The engine was fueled with no. 2 diesel pre-2007 certification fuel (Phillips Chemical Co., Borger, TX). Manufacturer-specified fuel properties included a 370-ppm sulfur content, 29% (by volume) aromatics, and a 47.6 cetane index. Engine lubricating oil (Rotella T 15w-40; Shell Oil Company, The Hague, Netherlands) was changed immediately before each 1-week exposure period. A bank of 500-W halogen lights pulled electrical current from the generator and provided a constant load that differed by the number of lights in operation. The engine operated at partial (55%) load with six lights and at high (100%) load with 11 lights. Engine load was defined as the number of watts drawn from the lights as a fraction of the total engine capacity.

The animal exposure chambers were 1 m^3^ whole-body inhalation chambers (H-1000; Hazelton Systems, Maywood, NJ) operated at a flow rate (250 L/min) that produced approximately 15 air exchanges per hour. We monitored and electronically recorded the temperature, relative humidity (RH), and flow using LabView software (version 6; National Instruments Corp., Austin, TX). Chamber temperature was maintained at 22–26°C.

We conducted DEE exposures 6 hr/day for either 3 (cardiovascular toxicity) or 7 (all other end points) consecutive days. We maintained exposure levels by adjusting the clean air dilution to a predetermined PM mass concentration, which was measured both in real time and as integrated over 30-min periods as described below. Concurrent exposures to HEPA- and charcoal-filtered air accompanied each DEE exposure. Other than exposure, we treated filtered-air control and DEE-exposed mice identically.

PM mass concentration was measured gravimetrically by sampling for 30-min intervals on 47-mm Pallflex filters (T60A20; Pall-Gelman, East Hills, NY) connected to the exposure chamber with a 20.3-cm-long × 0.6-cm internal-diameter probe connected to an aluminum in-line filter holder (In-Tox Products, Inc., Albuquerque, NM). We electronically measured pre- and postsample filter weights (MT5 balance; Mettler-Toledo, Columbus, OH). A static discharger was used before weighing filters to avoid interference from electrical charge. We characterized the composition of the exposure atmospheres in detail as reported (McDonald et al. 2004a).

*Animals.* We used two strains of mice previously established as disease or injury models in either compromised or healthy subjects. For assays of lung inflammation and resistance to infection, we used 8- to 10-week-old C57BL/6 male mice (Charles River Laboratories, Inc., Wilmington, MA) that were fed a normal ration (Teklad Rodent Diet; Harland, Madison, WI). For assays of HR and HR abnormalities, we used 8- to 12-week-old ApoE^–/–^ male mice (Jackson Laboratories, Bar Harbor, ME) that were fed a high-fat ration (Teklad JK050814; Harland) for 16 weeks before exposure. The ApoE^–/–^ mice are a susceptible model for cardiovascular toxicity because they have a markedly altered plasma lipid profile compared with normal mice and rapidly develop atherosclerotic lesions. All mice were provided water *ad libitum* and feed outside of exposure hours. All mice were housed in an Association for Assessment and Accreditation of Laboratory Animal Care–approved pathogen-free, rodent housing facility at controlled temperature (20–24°C) and humidity (30–60% RH) and quarantined for 2 weeks before use. Routine serological screens indicated an absence of mouse pathogens. The Lovelace Respiratory Research Institute Animal Care and Use Committee approved all procedures. The animals were treated humanely and with regard for alleviating suffering.

*Biological response assays.* Inflammatory signaling proteins (cytokines) were measured in homogenates of the right caudal and middle lung lobes (C57BL/6 mice, six per group). Immediately after sacrifice, the lungs were flash frozen. Before analysis, lungs were removed from the freezer and brought to room temperature. Lungs were homogenized for 1 min at full speed in a Tissuemizer (Tekmar, Mason, OH) and centrifuged for 5 min at 14,000 × n microfuge tubes and kept on ice. Heme oxygenase-1 (HO-1) and cytokines [tumor necrosis factor-α (TNF-α) and interferon-γ (IFN-γ)] were determined (two measurements for each cytokine for each sample) by enzyme-linked immunosorbent assays using commercially available analysis kits (Biosource International, Camarillo, CA). To normalize the cytokine measurements to total protein, we diluted the supernatants to 2 mg/mL in phosphate-buffered saline (PBS), and total protein was assayed by the Coomassie dye binding assay (Pierce, Rockford, IL) with bovine serum albumin as the standard.

Inflammation was assessed by the infiltration of inflammatory cells measured in bronchoalveolar lavage fluid. Lungs were lavaged three times with 1 mL PBS. The total number of leukocytes in the recovered lavage fluid was determined using a hemocytometer, and a cytocentrifuge slide was prepared and stained with Diff-Quick for evaluation of the differential cell types in the lavage fluid. These results are not reported because we observed no increase in lung inflammatory cells.

The effect of exposure on resistance to respiratory infection was studied in mice exposed to DEE diluted to approximately 250 μg/m^3^ PM. The study used a respiratory syncytial virus (RSV) model as previously described (Harrod et al. 2003). Immediately after the seventh day of exposure, groups of exposed and control C57BL/6 mice (*n* = 8 per group) were instilled intratracheally with 10^7^ plaque-forming units of cultured RSV. Parallel groups of exposed and control mice without infection were sacrificed immediately after exposure as controls for RSV pathology. Infected mice were housed individually in pathogen-free conditions in a Biosafety Level 2 animal facility for 4 days until sacrifice. One lobe of the lung was analyzed for the presence of RSV by densitometric analysis of virus-specific mRNA transcripts that were isolated by gel electrophoresis after gene amplification. Sections for histopathology were obtained from another lung lobe, stained with hematoxylin and eosin (H&E), and examined by a pathologist under a light microscope. Cross sections were obtained approximately 500 μm caudal to the junction of the mainstream bronchus. The condition of the airways and vessels was scored (0–4 scale) without knowledge of the treatment group.

The ApoE^–/–^ mouse model of cardiovascular response to air pollutants, including both its justification and its weaknesses in predicting human response to air pollutants, has been previously described (Campen et al. 2005). After 14 weeks on a high-fat diet, the mice were surgically implanted with radiotelemetry devices (DataSciences, Inc., St. Paul, MN) using a technique similar to that reported for rats (Campen et al. 2003) except that the telemeter remained subcutaneous, rather than being placed in the peritoneal cavity. All procedures were conducted under sterile conditions, and we allowed the mice 7 days to recover before acquisition of control data. The mice remained on the high-fat diet throughout the study period.

We exposed ApoE^–/–^ mice (*n* = 5 per group) for 6 hr/day for 3 consecutive days. The DEE concentration was 3.5 mg PM/m^3^. Signals from telemetry devices were captured by a digital acquisition system (Gould Ponemah Life Science Suite, Valley View, OH). HR was determined from beat-to-beat intervals, averaged over 5-min periods. For T-wave morphology analysis, a specialized ECG analysis package (ECGAuto; EMKA Technologies, Falls Church, VA) reviewed the collected waveforms. T-wave area was calculated as millivolts times seconds, based on the deviation from the isoelectric line defined as the onset of the Q-wave. We normalized values to each individual subject’s baseline values over the 24-hr period preceding exposure.

*Statistical analysis of data.* For each response category, analysis of variance (ANOVA) was used to evaluate mean differences between responses to DEE and concurrent filtered-air exposure. Levene’s test (Levene 1960) was used to evaluate the appropriateness of the standard ANOVA assumption of equality of variances among experimental group responses. These tests showed significant evidence of inequality of variances (*p* < 0.05) for all end points except lung histopathology. To alleviate this, a weighted least squares analysis (Neter et al. 1996) was employed using the reciprocals of the estimated variances in experimental groups as weights. *F*-test contrasts (Searle 1971) were used to compare differences in responses between emission and concurrent filtered-air control groups.

For HR and ECG, group data were tested by two-way ANOVA (time and DEE concentration as factors for whole-body ECG data; DEE and analyte concentrations for vessel assays). Bonferroni post hoc tests were used to determine specific differences. Probability values < 0.05 were considered significant. We used SAS (version 9.0; SAS Institute, Inc., Cary, NC) to perform all the statistical analyses.

## Results

**Figure 1:**
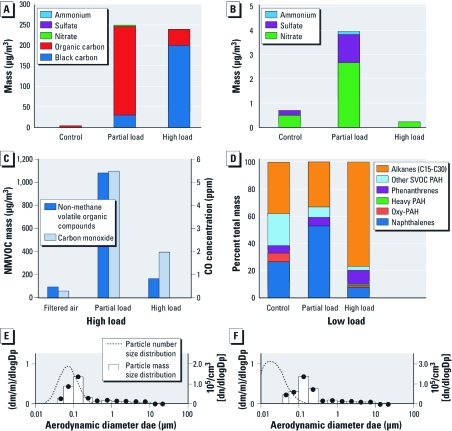
Mass composition of PM in the three exposure conditions. Abbreviations: Alkanes C15–C30, compounds with 15–30 carbon chain lengths; dae, aerodymanic particle size equivelant; dlogDP, differential log of particle size range; dm, differential mass; m, mass; Oxy-PAH, oxygenated polycyclic aromatic hydrocarbons. (*A*) The full mass composition of PM primarily dominated by carbon. Elemental (black) carbon accounted for most of the high-load exhaust, but its concentration was low in the partial-load operating condition. (*B*) Contributions from ammonium, sulfate, and nitrate to PM mass. Partial-load PM had much greater contributions from organic carbon, ammonium, sulfate, and nitrate material than did high-load PM. (*C*) Nonmethane volatile organic compounds (NMVOC) and CO for the three exposure conditions. The partial-load atmosphere consisted of much greater mass of both components than did the high-load or control atmospheres. (*D*) Relative proportions of major volatile organic compound classes, including semivolatile organic compounds (SVOCs) in the three experimental atmospheres. (*E* and *F*) PM mass size distributions did not differ between high- and partial-load modes, but the particle number size distributions did; the *y*-axis label [(dm/m)/dlogDp] refers to the differential mass, normalized to total mass within a specified particle size range. The black circles show the particle number size distribution.

*Composition of DEE.* A summary of the DEE composition and particle size is shown in Figure 1. The high-load operating mode produced exhaust with less carbon monoxide (CO) and less organic material than did the partial-load mode, which was dominated by organic carbon in both the gas and particle phases. The clean-air exposure contained low quantities of both gases and PM. PM mass size distributions did not differ between high- and partial-load modes, but the particle number size distributions did. The PM mass size distributions of both atmospheres had mass median diameters of approximately 0.15 μm. The particle number size distributions had medians of 0.085 μm at high load and 0.030 μm at partial load.

**Figure 2:**
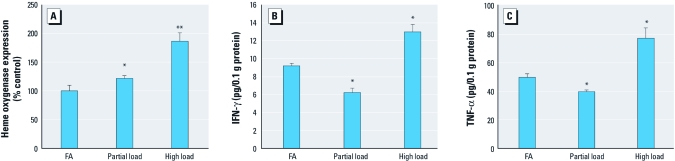
Changes in markers of pulmonary inflammation, HO-1 (*A*), IFN-γ (*B*), and TNF-α (*C*), for control [filtered air (FA)], partial-load DEE, and high-load DEE exposures (mean + SE). Inflammatory proteins were measured in homogenates of the right caudal and middle lung lobes (C57BL/6 mice, 6 per group). *p < 0.05 versus control, and **p < 0.05 versus control and partial load, by two-way ANOVA (*n* = 5 per group).

*Lung inflammation.* Figure 2 shows the concentrations of HO-1, IFN-γ, and TNF-α (normalized for total protein) in lung homogenate from C57BL/6 mice that were sacrificed immediately after DEE or filtered-air exposure (no infection). HO-1 was significantly up-regulated by both DEE exposures, although high load caused a stronger response than did particle load. Interestingly, we found significant (*p* < 0.01, ANOVA) reductions of both IFN-γ and TNF-α at partial load (relative to filtered-air concurrent controls) and significant (*p* < 0.01) increases at high load.

**Figure 3:**
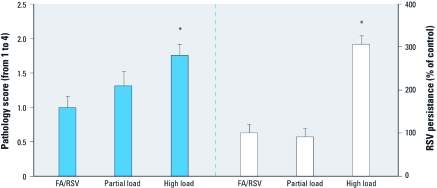
Pathology and viral clearance in C57BL/6 mice exposed to filtered air (FA; control), partial-load DEE, or high-load DEE, and RSV administration (mean + SE). *p < 0.05 versus control.

**Figure 4:**
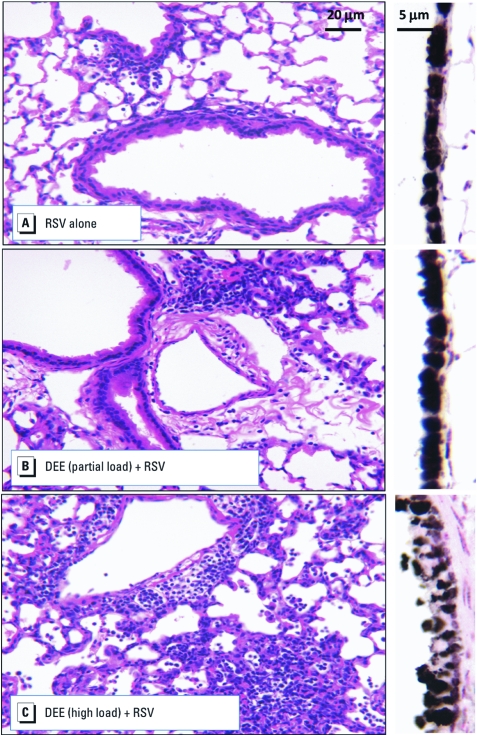
Lung pathology images of C57BL/6 mice exposed to filtered air (*A*), partial-load DEE (*B*), or high-load DEE (*C*) and RSV. H&E-stained lungs (left) show substantial increases in inflammation in mice exposed to both RSV and high-load DEE. Additionally, airway epithelial cells stained for Clara cell secretory protein (right) show more advanced pathology and differentiation after exposure to high-load DEE. *p < 0.05 versus control.

*Respiratory infection.* Figure 3 summarizes the lung responses of mice to RSV challenge, showing the histopathological score and the viral persistence, or the amount of RSV that was still present in the lungs 4 days after instillation. Exposure at high load caused a significant increase in pathological score compared with the clean-air controls, whereas exposure at partial load caused only a slight, nonsignificant increase. Exposure at high load also decreased clearance of RSV from the lungs (*p* = 0.002), but exposure at partial load did not reduce viral clearance. Histological inflammation was evident at baseline, due to RSV inoculation alone (Figure 4A). Exposure at high load considerably exacerbated inflammation and epithelial pathology (Figure 4B). Exposure at partial load increased inflammation somewhat but did not cause epithelial disruption (Figure 4C).

**Figure 5:**
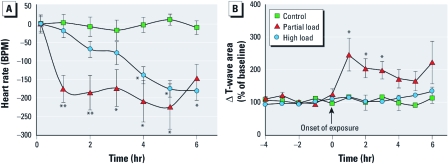
HR (*A*) and T-wave area (*B*) changes (mean ± SE) in ApoE^–/–^ mice exposed to filtered air, partial-load DEE, or high-load DEE. We obtained ECG signals continuously via radiotelemetry, and we derived data from the ECG and reported hourly data for analysis. Average HRs were 692 ± 13, 685 ± 40, and 648 ± 26 BPM for control, partial load, and high load, respectively. *p < 0.05 versus control, and **p < 0.05 versus control and high load, by two-way ANOVA (*n* = 5 per group).

*HR and ECG.* High-load DEE exposure induced a significant reduction in HR in ApoE^–/–^ mice (Figure 5), which was consistent with previous findings. The HR responses were gradual, achieving statistical significance during the fourth hour of exposure at roughly 100 beats/min (BPM) below control (Figure 5A). In contrast, exposure at partial load caused a nearly instantaneous significant reduction in HR, with average values in the first hour of roughly 175 BPM below control. Maximal reductions during the 6-hr exposure were roughly 215 BPM below baseline values at partial load. The HR returned to normal values within 1–2 hr after exposure at either load level (data not shown).

At partial load, T-wave alterations mirrored the HR changes, both in magnitude and in time to onset (Figure 5B). We saw a very rapid change in the T-wave in the first hour, resulting in a 150% increase in T-wave area. The high load caused no effects on T-wave.

## Discussion

The present study demonstrates that the operating load of a diesel engine has a significant effect on the composition of the exhaust and, not surprisingly, the relative toxic potency of inhaled exhaust. In a somewhat surprising contrast, lung responses in C57BL/6 mice were greater at high load, whereas cardiac responses in APOE^–/–^ mice were greater at partial load at the same PM mass concentration. We obtained these novel observations using fresh DEE and inhalation exposures of previously characterized models of toxicity. Exhaust produced under high load had a lower total organic content (both PM and gaseous), a higher content of elemental (black) carbon, and a slightly larger number-based particle size than did that produced at partial load. At both loads, the particles were in the ultrafine particle size range; however, the median aerodynamic diameter produced at high load was approximately 2.5–3 times that produced at partial load. Both atmospheres showed low sulfate concentrations (< 5% of PM), despite the use of fuel that had approximately 300 ppm sulfur content. Fuel after 2007 has < 15 ppm sulfur content, so the contribution of diesel to sulfur would decrease with more modern fuel relative to the pre-2007 fuel used in the present study. Exposure to high-load DEE produced lung responses in both the noninfected and infected mouse models. The responses were evident but clearly less at partial engine load. In some cases cytokines, including TNF-α, were suppressed after exposure to the partial-load atmosphere. TNF-α is classified as a “proinflammatory cytokine,” and a toxic effect is typically signaled by an increase. Because the physiological basis for the decrease in TNF-α at partial load is uncertain, this response is difficult to interpret.

The greater lung responses at high load suggest elemental carbon as a putative causal component. Elemental carbon was the only measured constituent that was present at a higher concentration at high load than at partial load. Of course, because we measured only select organic species in this study (polycyclic aromatic hydrocarbons, acids), some unmeasured organic compounds may be responsible or play a role in the observed effects. Furthermore, the higher CO levels in the low-load DEE may offer a protective effect similar to that described by Ryter and Choi (2010), where CO reduced inflammation in disease models when administered therapeutically. However, several epidemiology studies have shown associations between cardiovascular effects in people and exposure to black carbon as a proxy for traffic exposure (e.g., Zanobetti et al. 2010). Although those studies do not typically single out the toxicity of black carbon explicitly, the strong association lends plausibility that it may itself cause biological effects. A common misconception regarding the composition of elemental carbon is that it is analogous to “carbon, elemental,” which is often considered benign with respect to the biological end points studied here. “Elemental carbon” is actually a term defining the most light-absorbing portion of carbon, which is also most commonly defined functionally by its measurement as part of thermal–optical measurement methods (e.g., Chow et al. 2005). As described by Setyan et al. (2009), elemental carbon can contain functional groups and can have varying surface chemistry. As an example of the potential for elemental carbon with enhanced surface chemistry to interact with biological proteins, Seagrave et al. (2004) showed that elemental carbon with differential functional groups and surface chemistry can result in different interactions with inflammatory cytokines. An important point to consider is that if elemental carbon plays a role in the health effects of DEE, it is noteworthy that the diesel particle trap technology implemented to meet the U.S. 2007 on-road emission standards effectively removes most elemental carbon from exhaust. This was evident in our previous study, where we used the engine system described here coupled with a diesel particle trap and showed that elemental carbon was removed (and health effects were also reduced) (McDonald et al. 2004c).

The effects of DEE on the clearance of pathogens or susceptibility to infection have been reported in previous publications (Harrod et al. 2003; Reed and Berger 2006). We conducted those studies on a different engine system with a variable duty cycle. Indeed, both DEE and carbon have been shown in previous studies to have an effect on clearance of RSV. We have reported studies in which DEE either had an effect on RSV clearance (Harrod et al. 2003; McDonald et al. 2004c) or did not have an effect on RSV clearance (Reed and Berger 2006). Those studies used different DEE systems. In addition, the pathogenicity of the two lots of RSV used in the two studies differed, which may explain the apparent disparity of results. Further investigations confirmed the importance of viral passage in its pathogenicity, because we determined the characteristics and lineage of the virus to be important determinants of responses. In the present study, we treated all groups with RSV from the same viral passage and lineage conditions. As a result, the differences observed between high and partial load are believed to relate to differences in exhaust composition and not to differences in the virus.

We have previously established that high levels of DEE can induce bradycardia and T-wave alterations in mice and that ApoE^–/–^ mice were more susceptible than their genetic background, C57BL/6 mice (Campen et al. 2005). These effects appear to be highly congruous with data from controlled diesel exposures with coronary artery disease patients, wherein significant T-wave abnormalities develop during combined exercise and exposure (Mills et al. 2007). The effects in previous rodent studies were primarily linked to the gas-phase constituents, as evidenced by the robust bradycardic responses elicited by an atmosphere with the particles removed by a ceramic filter. The present study is therefore consistent, showing a greater cardiovascular response caused by the partial-load atmosphere, which was characterized as having greater levels of gaseous pollutants. The nature of the cardiac responses was not fully characterized, but earlier work suggested that the bradycardia and bradyarrhythmias (observed in the present study but not quantified) were a result of vagal stimulation (Watkinson et al. 2001). The T-wave morphology, however, was a finding seemingly unique to models of cardiac disease, namely, the spontaneously hypertensive rat (Kodavanti et al. 2000) and the ApoE^–/–^ mouse (Campen et al. 2005). We postulate that the high-fat diet regimen advances the natural vascular pathology in this model sufficiently to predispose the heart to occlusive ischemic events, similar to those reported by Caligiuri et al. (1999).

## Conclusion

The present study highlights the role of DEE composition in driving divergent pathologies. These findings have implications for the design and interpretation of experiments involving experimental exposures of humans and animals to DEE. Using a difference of only 55% and 100% rated load, this study made clear that engine load alone can alter the nature and magnitude of lung and cardiac responses. It can be speculated, if not yet determined, that idle versus partial or full load might induce greater differences in response and that any other factor affecting the composition of DEE can also affect biological responses and cause results to differ among studies. Few would argue this point, but little attention has been given to the composition of DEE in reporting and comparing studies. Because real-world exposures result from a spectrum of engine types operated in a wide range of modes using different fuels, lubricating oils, and exhaust after-treatment, it is probably not useful to contemplate establishing a “standard” DEE for experimental exposures. Together, however, the present and previous results clearly indicate the importance of characterizing the composition of DEE used in laboratory exposures to a greater extent than has been typical of the literature to date. The present results demonstrate clearly that PM mass concentration alone is an inadequate metric for comparing results among studies or for estimating hazards or risks from exposure–response slopes using results from studies conducted under different conditions of engine type and operation.
